# Data of the patterns of youth local brand product consumption through online shopping

**DOI:** 10.1016/j.dib.2019.103723

**Published:** 2019-03-07

**Authors:** Bagong Suyanto, Henri Subiakto, Koko Srimulyo

**Affiliations:** a Department of Sociology, Faculty of Social and Political Sciences, Airlangga University, Indonesia; b Department of Communication, Faculty Social Sciences and Political Sciences, Airlangga University, Indonesia; c Department of Library and Information Science, Faculty of Social and Political Sciences, Airlangga University, Indonesia

**Keywords:** Online shopping, Local brand, Youths, Consumers

## Abstract

This article presents data on the shift in urban youth consumption patterns from offline shopping to online shopping, and data on the tastes of urban youths towards local branded products marketed through online shopping. The data in this article were obtained from the results of field studies in five cities and regencies, namely: Surabaya City, Malang City, Kediri City, Sidoarjo Regency and Jember Regency. The number of respondents surveyed was 500. The research data can be used by researchers who are interested in studying lifestyle and consumption behaviour of urban youth. The data on patterns of consumption behaviour of young people towards local brand products can also be used as a reference for local brand business people to develop their business development strategies if they are targeting at young people.

**Table:** Specifications table

Subject area	Social Sciences
More specific subject area	Consumer behaviour and youth
Type of data	Texts and tables
How data was acquired	Researchers conducted surveys and in-depth interviews
Data format	Raw and analysed
Experimental factors	The data in this research was obtained from individual interviews with 500 youths aged 14–21 years old. Surveys were guided by previously prepared questionnaires. Respondent criteria: (1) youths aged 14–21 years old and (2) making online purchase(s) of local brand product(s) at some points within the last six months. In-depth interviews were also conducted with 50 urban youths.
Experimental features	Surveys were conducted to acquire data on youths' taste for local brand products and factors encouraging urban youths to consume local brand products. Surveys were conducted to explore data on the patterns of youths' consumer behaviour towards local brand products marketed online. In-depth interviews were conducted to collect information on the appeal of online shopping and youths' reasons for consuming local brand products.
Data source location	City of Surabaya, City of Malang, City of Kediri, Regency of Sidoarjo, and Regency of Jember in the Province of East Java
Data accessibility	Data is included in this article

**Table:** 

**Value of the data** 1. This research presents findings on youths' taste and pattern of consumer behaviour towards local brand products that can be taken into consideration by other researchers interested in reviewing consumer behaviour patterns of youths in the post-modern era. 2. This research results also present data on the actions taken by youths when being disappointed with online transactions that fell short of their expectation. 3. Government agencies intending to support the development of local brand products may use the data presented in this research as a reference for formulating local brand entrepreneur empowerment programmes. 4. Data on the patterns of youths' consumer behaviour towards local brand products can be used as a reference by local brand entrepreneurs in developing strategies for the development of their businesses targeting youths.

## 1 Data

The data presented is not only about the variety of goods purchased through online shopping, but also the experiences of young people when buying goods through online shopping. This data article also presents data about what local brand products which are consumed by young people, and how the advantages of shopping online can enable consumers to compare what they buy as the main attraction for young people to do online shopping. The data were based on the interviews conducted with 500 youths, who were potential consumers of local and foreign brand products. As many as 65 per cent of respondents were female, while the remaining 35 per cent were male. Respondents were within the age range of 14–21 years, most of whom were 20–21 years of age (43.2 per cent). The majority of the respondents were unemployed students who were still dependent on their parents. Out of the 500 respondents interviewed, 33 per cent were 16–17 years of age, 15 per cent were 18–19 years of age and 8.8 per cent were 14–15 years of age. As many as 55.4 per cent of respondents were middle school and high school students, and 35.6 per cent were university students.

## 2 Design, materials, and methods

The data in this research was collected from interviews with youths, who were potential consumers of industrial-cultural products and were part of the net generation with familiarity with the use of information technology for making transactions in an economic way. The number of urban youths, who were potential consumers of both local and international brand products, interviewed was set at 500.

The following aspects were adhered to for the interview process in order to fulfil the ethical considerations. In the process of collecting the data in the field, this study was assisted by five surveyors who were first trained and given an understanding of research ethics. At the beginning of the interview, the surveyors had informed the respondents that they would keep the information provided by the respondents confidential. The respondent's identity will not be revealed, and the information that the respondents put forward will only be processed along with other information obtained from interviews (see Table 1).

**Table 1:** Locations and number of respondents of research.

Subdistricts/Villages	Districts	City/Regency	Number of Respondents
Kertajaya Babatan	Gubeng Wiyung	City of Surabaya	100
Klojen Lowokwaru	Klojen Lowokwaru	City of Malang	100
Kampung Dalem Burengan	Kota Pesantren	City of Kediri	100
Gedangan Pucanganom	Gedangan Sidoarjo	Regency of Sidoarjo	100
Krajan Timur Jemberlor	Sumbersari Patrang	Regency of Jember	100
**Total**			**500**

Primary data was gathered and explored from regions that were developing and had developed into metropolitan areas in the Province of East Java. The locations purposively selected for this research consisted of five big cities/regencies in the Province of East Java, namely the City of Surabaya, the City of Malang, the City of Kediri, the Regency of Sidoarjo and the Regency of Jember. These five areas were chosen because they are the largest urban areas in East Java Province which are mostly inhabited by urban young people who are beginning to experience a shift in consumption patterns from offline to online patterns. From each city/regency, 100 youths were purposively selected. The respondents interviewed had to meet these criteria: (1) they were aged 14–21 years and (2) they bought local brand products online at some points within the last six months. In the process of finding the respondents, we selected the young people from 10 sub-districts and 10 villages of each cities/regencies. Consequently, a total of 500 young people interviewed, and they were part of the internet generation who were used to using information technology and accessing the internet.

The primary data required for this research was collected not only through direct interviews guided by a set of structured questionnaires previously prepared but also through in-depth interviews. These in-depth interviews were deemed necessary because this study reviewed not only how the pattern of urban youths’ behaviour shifted to the pattern of online shopping but also their construction of local brand products. In-depth interviews were conducted with 50 young people who had interesting experiences in consuming cultural industry products through online shopping, especially the products that are included as local brands. The in-depth interviews were conducted with a guided interview questions that had been prepared in advance.

All data from the results of interviews with the 500 respondents were edited and processed with the SPSS (Statistical Package for Social Sciences) program. The data are displayed in the form of frequency tables to see the trends and behaviour patterns of young people in consuming local brand products.

### 2.1 Shift of consumption pattern (offline to online)

In the post-modern era, the lifestyles developed by youths are reflected in not only what they consume but also how they consume industrial-cultural products [1]. Urban youths also have the hegemony for consuming capitalist products [2]. In major cities, youths’ shopping activities are not only conducted offline but also online. Widespread credit card ownership not only offers convenience for payments but also opens an opportunity for urban youths to purchase assorted products online [3].

There is an indication that the consumption pattern developing in the community, especially among youths, is shifting. Although most youths favour spending leisure time shopping while hanging out at department stores, there is an indication that some others start to find online shopping more pleasing. The variety of convenience offered and multitude of advantages accessible to youngsters if they do online shopping have somewhat shifted their consumption pattern.

**Fig. 1:**
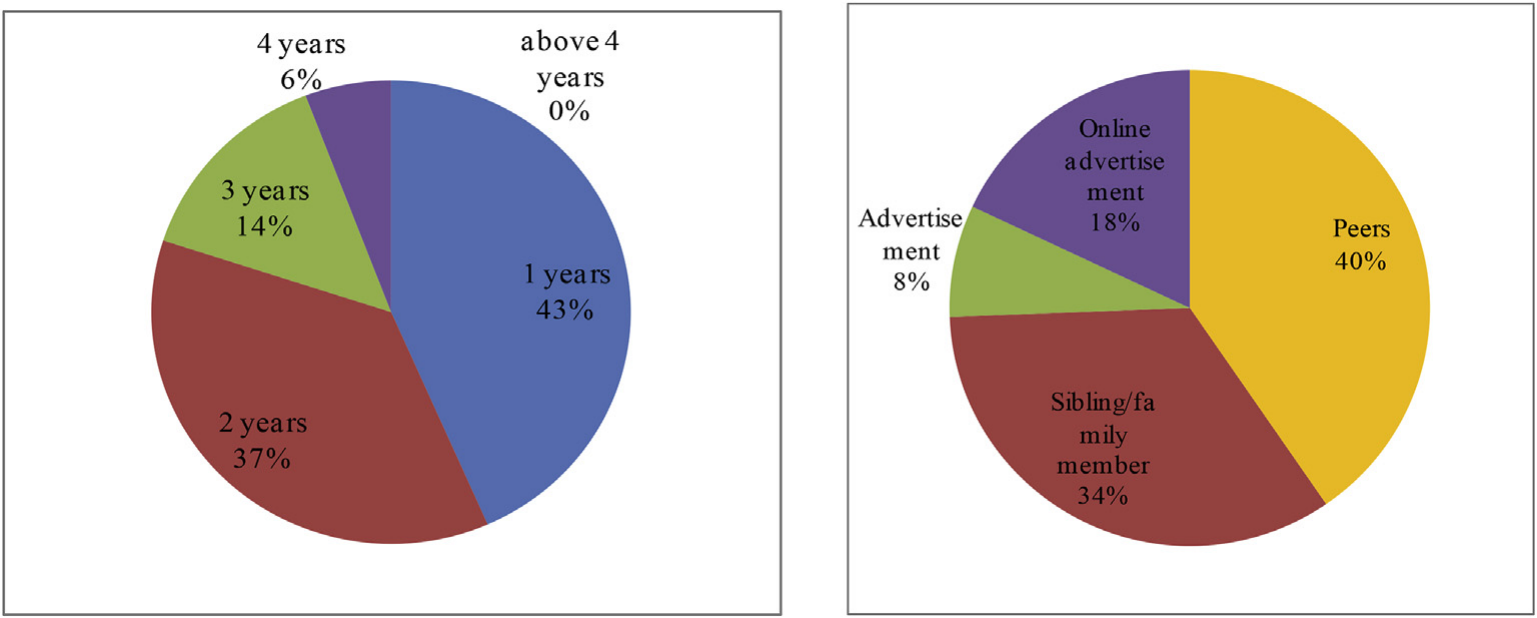
The first person/thing inducing respondents to do online shopping.

**Fig. 2:**
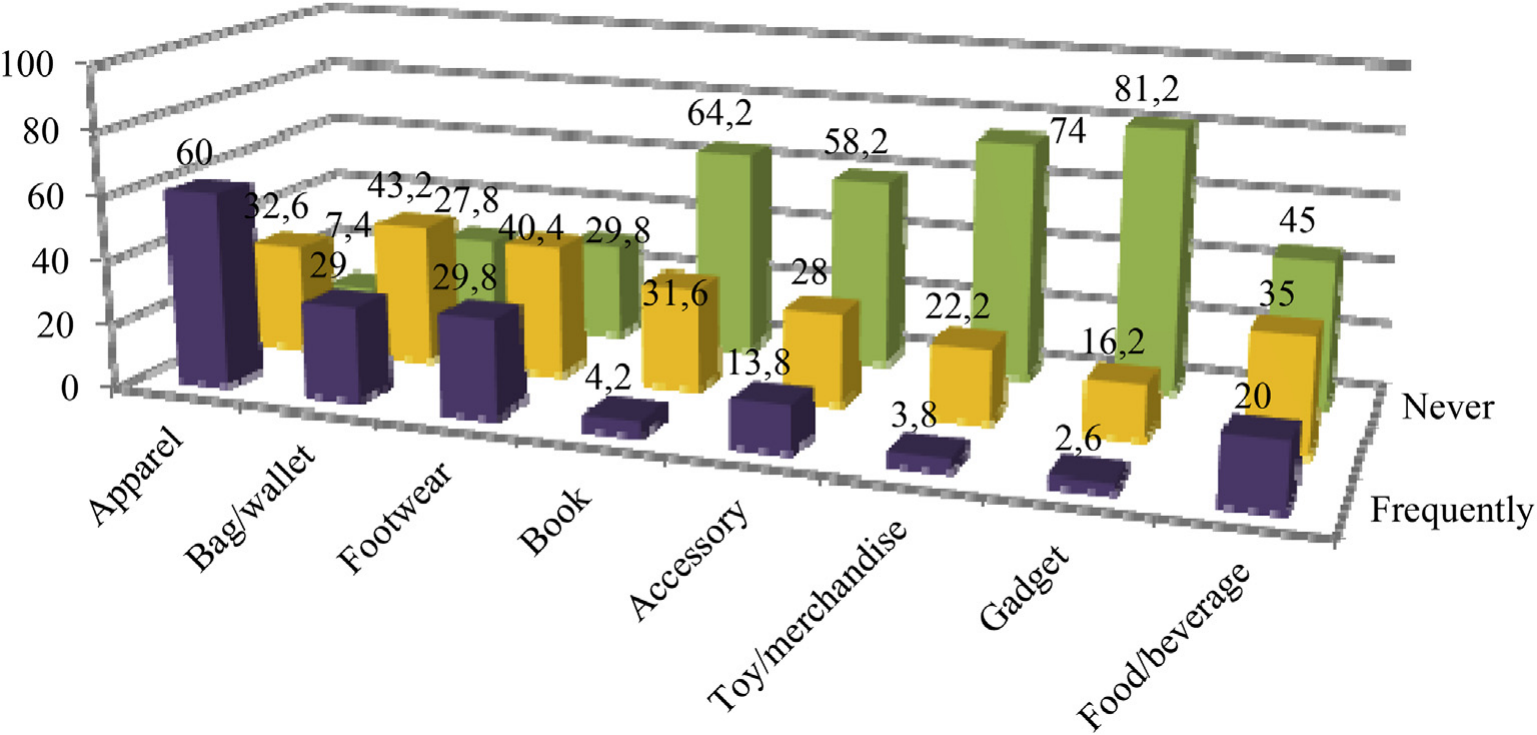
Items bought by respondents by shopping online.

**Fig. 3:**
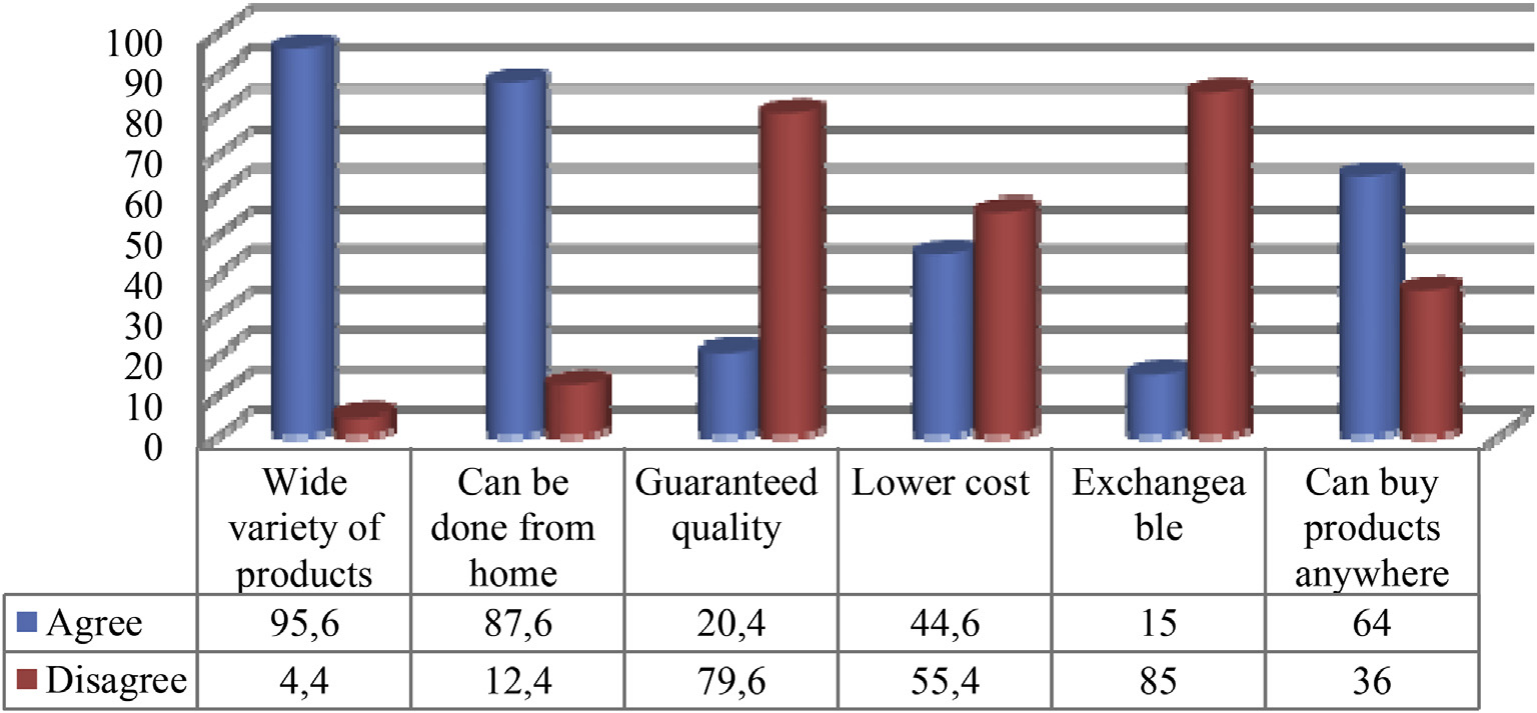
The appeal of buying products online.

**Fig. 4:**
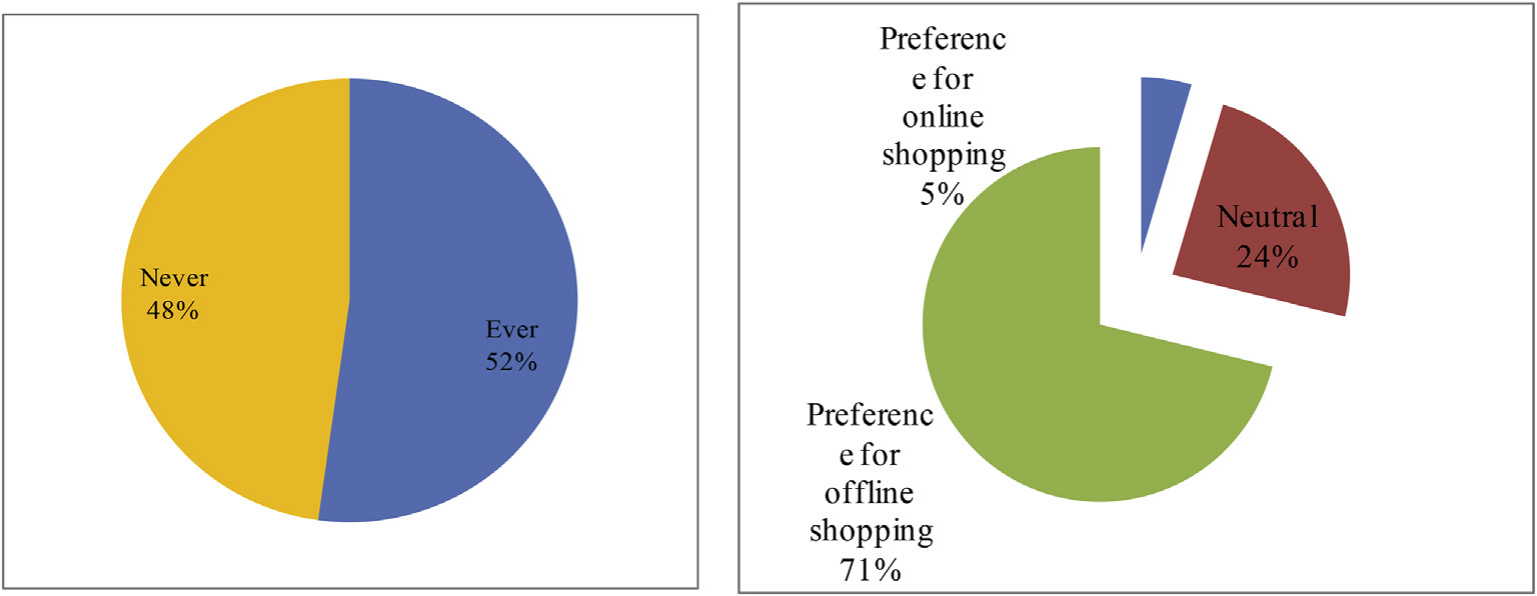
Experience of purchasing products online.

**Fig. 5:**
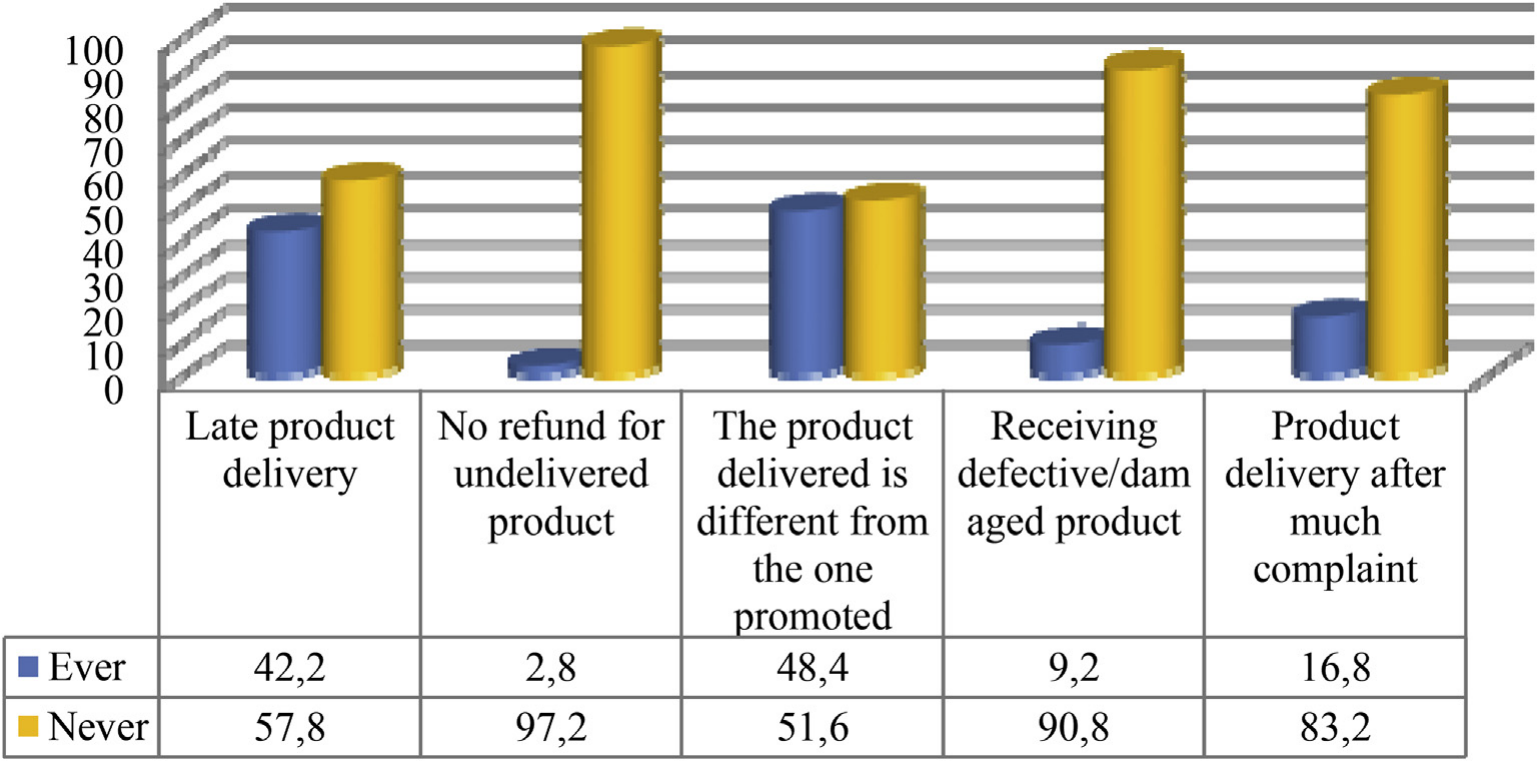
Things experienced by respondents when purchasing products online.

**Fig. 6:**
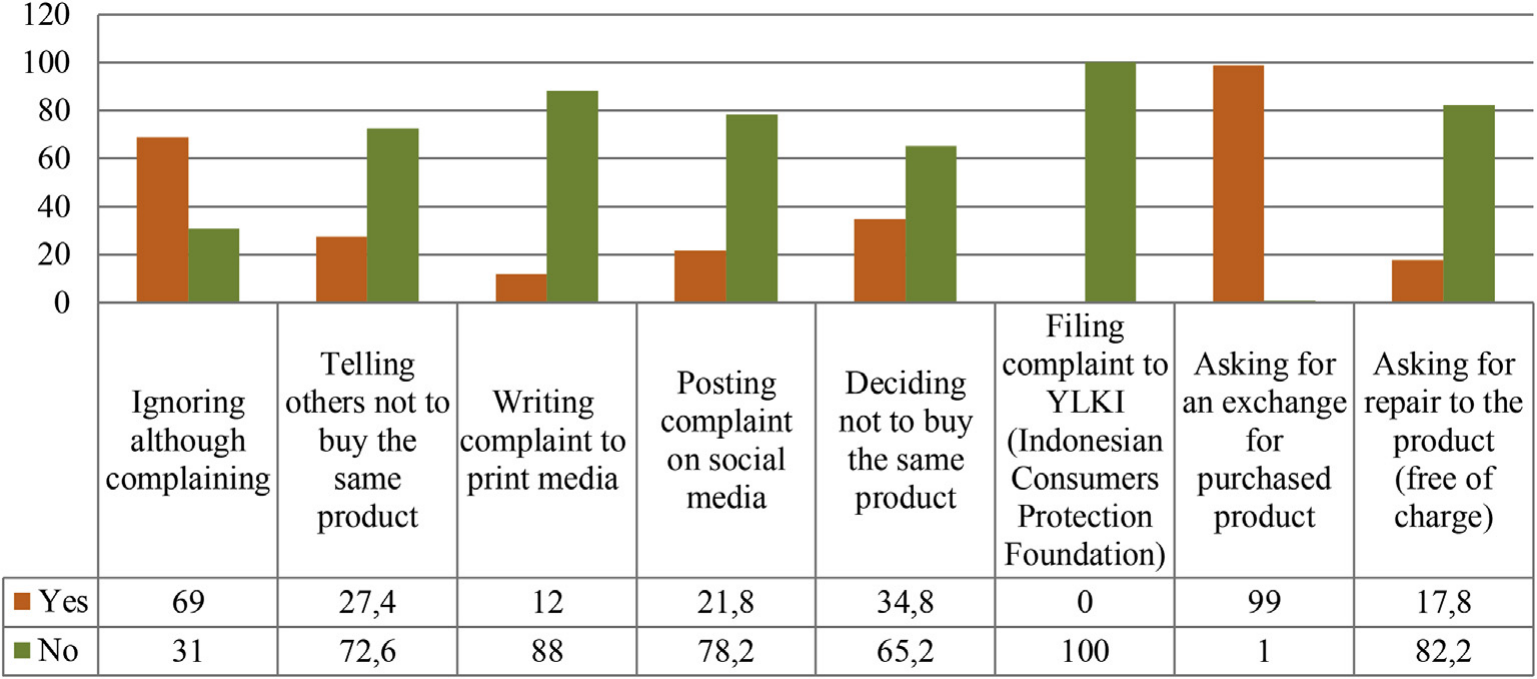
The actions taken by respondents when being disappointed in/incurring loss from online shopping.

**Fig. 7:**
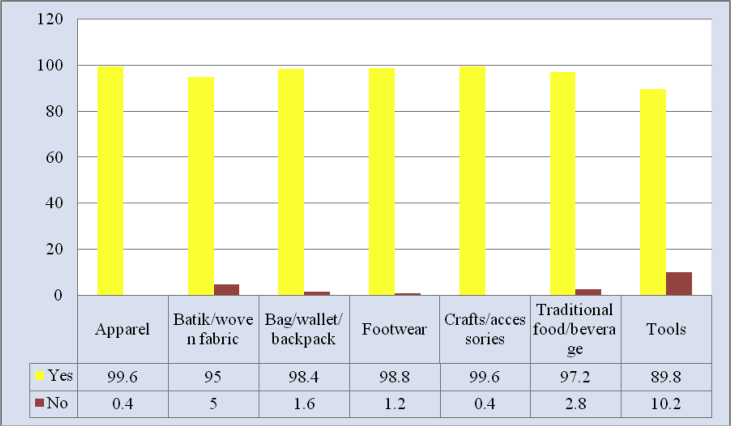
Identifiable local brands marketed online.

**Fig. 8:**
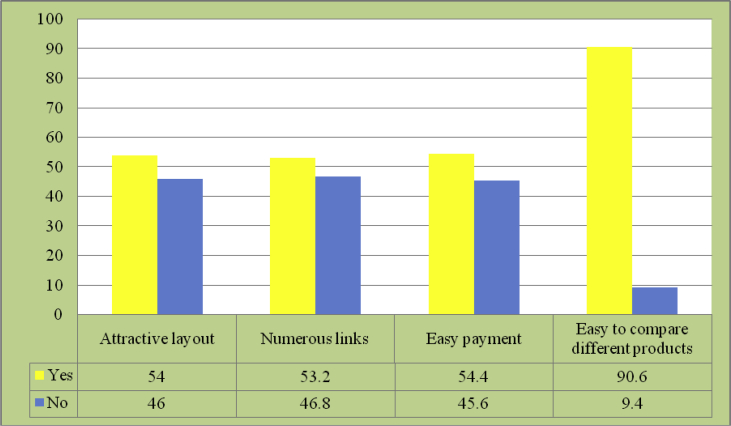
Attractive points of purchasing products online.

**Fig. 9:**
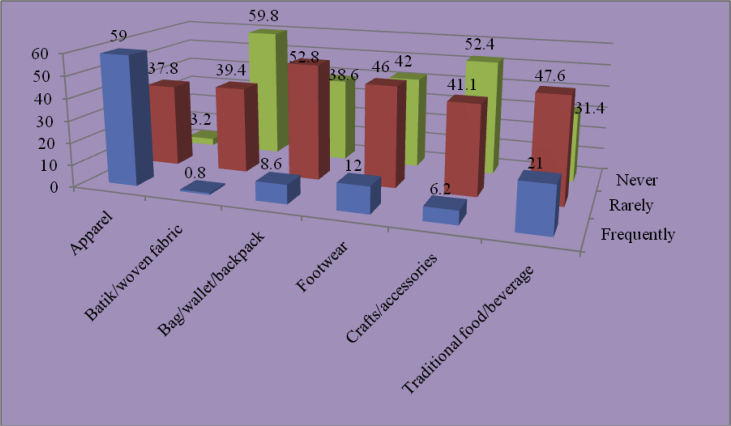
Local brand products frequently bought by respondents online.

The convenience offered in economic transactions, notably after the advent of online shopping, has exposed youths as consumers to a wide range of shopping options. In current digital era, online shopping often lures buyers with the absence of the need to go all the way to stores and spend money for transport just to purchase something. They just need to scroll down a web page and click on a link on their laptop screen to view and own any product (Figs. 1–9).

In different developed countries, the convenience of online shopping has long been felt. People in developed countries have been doing online shopping in the past decade. However, among Indonesian young people, online shopping is still fairly new. This study found that Indonesian youths have just been doing online shopping for the last 1–4 years. Most of the respondents (43.4 per cent) have become acquainted with online shopping within this past year. Out of 500 youths investigated, 36.6 per cent said that they have been doing online shopping in the last 2 years. Only 14.2 per cent of respondents revealed that they have been doing online shopping in the last 3 years and 5.8 per cent in the last 4 years.

Most youths were induced to engage in online shopping for the first time by their peers. As fellow youths and part of the net generation, school peers or playmates were the ones introducing the advantages and convenience of online shopping to respondents. To buy any product anywhere, respondents said that it is no longer a problem today. To purchase books from Yogyakarta, for example, youths domiciled in Surabaya do not need to bother to go all the way to Yogyakarta. It just takes them some clicks on the web of a well-known bookstore in Yogyakarta to order, purchase and take a hold of books they desire in just a couple of days. Out of 500 interviewed youths, 34 per cent said that the ones introducing online shopping to them were their own siblings or family members. The remaining 18 per cent got to know online shopping from ubiquitous advertisements in the virtual world.

The items bought by youths online vary from time to time. The items most frequently bought online were clothing (60 per cent). Others were bags/wallets (29 per cent), footwears (29.8 per cent) and accessories (13.8 per cent). As many as 4.2 per cent of respondents revealed that they bought books online. Interestingly, 20 per cent of respondents admitted that they also bought food/beverage online.

In the case of gadget, 81.2 per cent of respondents said that they had never done online shopping to buy one. Only 2.6 per cent admitted that they bought gadget online at some points in time. Some of the respondents expressed their concern about the security of buying gadget online. Peers’ accounts and mass media reporting on fraudulent gadget online trading have discouraged youths from making a purchase of gadget online.

Purchasing goods by shopping online, according to youths, is not less appealing than buying directly at offline stores. Although some youths investigated in this research still favoured buying goods offline, particularly when they needed the goods immediately, they still found some advantages of online shopping in some cases. One of the appeals of buying products online is the wide variety of products offered, even in an almost infinite amount (95.6 per cent). Moreover, 87.6 per cent of respondents said that online shopping was appealing to them because they could engage in it while relaxing at home or in the bedroom. As many as 64 per cent respondents shared that they found online shopping appealing because it allowed them to purchase goods from any place. Unlike department stores from which costumers’ purchase is only limited to displayed items, online shopping enables them to explore the virtual world infinitely. With only a laptop or a hand phone and Internet access, youths can pick a product and make a purchase just by playing with their fingers, viewing the products they desired and making a purchase.

In addition to the advantages above, some youths also stated that what makes online shopping appealing is the guaranteed quality of the products (20.4 per cent), lower cost (44.6 per cent) and even the opportunity to exchange items (15 per cent). Although they can also exchange items at department stores or other stores, respondents admitted that they were reluctant to meet shopkeepers who often pulled a scowling face when they had to make a direct return. When making a purchase online, the lack of the need to face sellers or shopkeepers in person offered comfort to some respondents. The flexibility to choose and purchase goods and the opportunity to return the goods combined were appealing to respondents.

In many cases, online shopping has many advantages. But it does not directly translate that youths doing online shopping are never left with disappointment. Out of the 500 youths investigated, more than a half (52.2 per cent) admitted that they had incurred damage from engaging in online shopping at some points in time. Different from the practice of online shopping in developed countries which is constantly under strict supervision and with consumers' rights truly assured, the practice of online shopping at home is admittedly still tainted with fraudulence and pervasive sellers’ unprofessionalism. Despite enjoying online shopping benefits, youths still engaged in online shopping with a bit of suspicion for these reasons.

Some bitter experiences youths gained when doing online shopping can definitely leave them feeling upset. As many as 42.2 per cent of respondents admitted that they experienced delayed product delivery. Meanwhile, 48.4 per cent of respondents had ever received products that did not suit the one pictured in the promotion. There were even 9.2 per cent of respondents who received defective or damaged products. Worse yet, 2.8 per cent of respondents fell victims of fraud. They had made a payment, but the products ordered had never reached them.

Reflecting on such unpleasant experiences mentioned above, for the time being, 71.2 per cent of respondents admitted that they preferred offline shopping to online shopping. Only 4.6 per cent of respondents favoured online shopping, while the remaining 24.2 per cent did not find any difference between online shopping and offline shopping. In some instances, namely when the products of interest were only available outside the city, youths found online shopping more interesting. However, if they sought to go shopping and hang out at the same time, offline shopping was a better option for them.

What do youths do when they are disappointed in or suffer from loss from online shopping? When this question was posed, most respondents (69 per cent) answered that they just ignored it although they still complained. They did nothing, especially when they realized that they had fallen victim to a fraud. But if the respondents were informed that the sellers were bona fide, nearly all of them (99 per cent) would ask for an exchange for the purchased products if defects were found or if the products received differed from the ones promoted. As many as 34.8 per cent of respondents even decided not to repurchase the same products from the same sellers. As many as 21.8 per cent of respondents stated that they posted complaint on social media, and 12 per cent of respondents even wrote complaint to print media to draw attention and tell other buyers to exercise caution.

### 2. 2 Online shopping and local products

The virtual world nowadays offers the ease to view not only prestigious global products but also local products. Local brands marketed online that are popular among youths abound. It can be said that nearly all local brands offer varying lines of products, starting from apparel (99.6 per cent), to batik or woven fabric (95 per cent), to crafts (99.6 per cent), to bag, wallet and backpack (98.4 percent), among others. This means that when youths surf online, they are exposed to not only imported product offers but also local products that are equally reputable.

Online shopping presents an advantage, in that buyers can compare a variety of available products with ease (90.6 per cent). While at offline stores buyers are only exposed to displayed products, they have a greater access to browsing through products offered across the nation, or even the globe, as well as various brands there are in the market when doing online shopping.

As many as 53.2 per cent respondents stated that online shopping was interesting because of the many links allowing them to view products they desired. Meanwhile, 54.4 per cent of respondents said that online shopping's attractive point was its easy payment. Equipped with only credit card or debt card, anyone will find it easy to do online shopping.

Local brand products frequently purchased by youths online mostly were apparel (59 per cent). There were hardly any youths buying batik or woven fabric; only 0.8 per cent of respondents admitted to buy batik or woven fabric. According to some respondents, it was their parents who often bought batik or woven fabric. Young people predominantly preferred fashionable apparel suiting their lifestyles.

Another product frequently purchased by youths was footwear. As many as 12 per cent of respondents said that they often bought footwear online, while 46 per cent others said that they only occasionally did so. Respondents also fairly often bought food and beverage online (21 per cent). Regardless of which products youth bought online, it is worth noting that today's era has been changing, and the development of information technology and Internet has strengthened consumers' desire, with no exception of urban youths.
