# Supplementary material for: Data of the patterns of youth local brand product consumption through online shopping
Source: Data Brief. 2019 Mar 7;23:103723. doi: 10.1016/j.dib.2019.103723 (PMC6660617; doi:10.1016/j.dib.2019.103723)
Supplement: Supplementary file 1 — Multimedia component 1 [file mmc1.pdf]

AUTHOR DECLARATION

We wish to confirm that there are no known conflicts of interest associated with this publication and there has been no significant financial support for this work that could have influenced its outcome.

We confirm that there are no other persons who satisfied the criteria for authorship but are not listed.

We confirm that we have given due consideration to the protection of intellectual property associated with this work and that there are no impediments to publication, including the timing of publication, with respect to intellectual property. In so doing we confirm that we have followed the regulations of our institutions concerning intellectual property.

We understand that the Corresponding Author is the sole contact for the Editorial process (including Editorial Manager and direct communications with the office). He is responsible for communicating with submissions of revisions and final approval of proofs. We confirm that we have provided a current, correct email address which is accessible by the Corresponding Author and which has been configured to accept email from bagong_fisip@yahoo.com.

Indonesia, 24 October 2018

Bagong Suyanto
(Corresponding author)

Henri Subiakto

Koko Srimulyo
